# Age–period–cohort analysis of epidemiological trends in pelvic fracture in China from 1992 to 2021 and forecasts for 2046

**DOI:** 10.3389/fpubh.2024.1428068

**Published:** 2024-07-08

**Authors:** Qingsong Chen, Tao Li, Hong Ding, Guangbin Huang, Dingyuan Du, Jun Yang

**Affiliations:** ^1^School of Microelectronics and Communication Engineering of Chongqing University, Chongqing University Central Hospital (Chongqing Emergency Medical Center), Chongqing, China; ^2^Department of Traumatology, National Regional Trauma Medical Center, Chongqing University Central Hospital (Chongqing Emergency Medical Center), Chongqing, China; ^3^Department of Orthopedics, Chongqing University Central Hospital, Chongqing Emergency Medical Center, Chongqing, China

**Keywords:** pelvic fracture, joinpoint regression, age-period-cohort analysis, incidence, years lived with disability, Nordpred model

## Abstract

**Background:**

This study explored the epidemiological trends in pelvic fracture (PF) in China from 1992 to 2021, analyze their relationships with age–period–cohort (APC) factors, and predict the trends of PF from 2022 to 2046.

**Methods:**

Incidence and years lived with disabilities (YLDs) of PF among sexes in China from 1992 to 2021 were obtained through the 2021 Global Burden of Disease (GBD) database. Trends in the incidence and YLDs were described, and a joinpoint regression model was used. The APC model was used to explore the effects of age, period, and cohort on the incidence and YLDs. Nordpred forecasted the incidence and YLDs in China from 2022 to 2046.

**Results:**

In 2021, there were an estimated 0.63 million incidence cases and 0.33 million of YLDs, respectively. The number and age-standardized rate (ASR) of incidence and YLDs were both gradually increased. The average annual percent change (AAPC) in incidence and YLDs for men were 0.26% and −0.17%, respectively. For women, the AAPC values for incidence and YLDs were −0.03% and −0.57% (*p* < 0. 001), respectively. The relative risk (RR) of PF increases with age, with the lowest risk in those aged 10–14 years for incidence and aged 1–4 for YLDs and the highest risk in those aged >95 years for incidence and aged 90–94 years for YLDs. The period effect showed a totally increase in the risk across the general, male, and female populations. Cohort effects indicated a totally significant decline for both incidence and YLDs. The predicted incidence and YLDs of PF in China from 2022 to 2046 showed an initial rise, followed by a decline, with 2029 and 2034 being the turning point for incidence and YLDs, respectively.

**Conclusion:**

The characteristics of pelvic fracture incidence and YLDs in China are complex. Thus, primary prevention measures must be strengthened. Raising awareness about osteoporosis prevention, enhancing public health education, and promoting good dietary and hygiene habits are appropriate preventive measures for PF in China.

## Highlights

- The incidence and YLDs of pelvic fractures both exhibited a sharply decreasing trends followed by a gradual increase from in China.
- Generally, the pelvic fracture’s incidence and YLDs are associated with age increasing, but demonstrating fluctuations in specific age groups.
- From 2022 to 2046, the rate of incidence and YLDs of pelvic fracture in China are projected to initially rise and then decline, which reaching a peak at 2029 for incidence and 2034 for YLDs.
- The future trend of pelvic fractures in China is primarily associated with osteoporosis, which may be closely linked to population aging.

## Introduction

Pelvic fracture is severe fractures commonly associated with high-energy traumas such as traffic accidents, falls from a height, and crush injuries. They are among the most complex injuries encountered in trauma care (1, 2). In trauma centers, approximately 10% of patients with severe trauma sustain PF (3). The overall incidence of PF is increasing annually because of inevitable factors such as population aging, with nearly exponential growth observed (4). The mortality and disability rates from PF are high, particularly when the injury severity score is >25 points, often accompanied by injuries to other organs. This can lead to complications such as hemorrhagic shock and traumatic coagulopathy, further endangering the patient’s life (5). Pelvic fractures are severe injuries associated with high mortality rates and poor prognoses, particularly common among older adult patients. With the increasing aging population, the incidence of fragility pelvic fractures has significantly risen, especially among older adults. Fragility pelvic fractures are those related to osteoporosis, often occurring with minor trauma or spontaneously. Studies indicate that the incidence rate of fragility fractures ranges from 35.5 to 121.2 per 100,000 persons annually. The ambulatory ability of patients with fragility pelvic fractures significantly declines by about 44.4% within 1 year post-injury, with 1-year and 5-year mortality rates of 15.4 and 39.9%, respectively (6). Additionally, a study in Sweden reported that between 2001 and 2016, the incidence of pelvic and acetabular fractures increased from 64 to 80 per 100,000 persons annually. Pelvic fractures predominantly occurred in women (74%), while acetabular fractures were more common in men (58%). This study further confirmed the high incidence of pelvic and acetabular fractures among the older adults and their profound impact on patients’ quality of life (7). Women aged 80 and above are particularly at high risk. The study found that patients with pelvic ring fractures had poor functional recovery and significantly reduced walking ability post-injury, with high mortality rates. These findings highlight the importance of prevention and early intervention in reducing incidence rates and improving prognoses (8).

After pelvic fracture, patients often need prolonged bed rest, but this can lead to complications such as aspiration pneumonia and deep vein thrombosis, which can severely impact daily life and even threaten the patient’s health (9). Moreover, the impact of pelvic fractures is long-term rather than temporary, significantly affecting the patient’s quality of life over time (10). The prevention of pelvic fractures is of great clinical and social importance. With the aging population, the incidence of pelvic fractures is increasingly related to osteoporosis. Therefore, targeted measures to prevent osteoporosis, such as calcium and vitamin D supplementation and a healthy diet, are crucial for preventing osteoporotic fractures (11). Additionally, the treatment costs for pelvic fractures are high, including emergency treatment, surgical fees, hospitalization costs, and subsequent rehabilitation expenses, imposing a heavy economic burden on the healthcare system (12). Moreover, road traffic accidents and falls from a height are still the leading causes of PF (13). In China, epidemiological research on PF is limited, and no studies have used the age–period–cohort (APC) model to estimate the overall trends and parameters of the incidence and years lived with disability (YLDs) of PF from 1990 to 2021 to analyze the epidemiological characteristics (14). The joinpoint regression (JPR) model was used to quantitatively estimate and analyze changes in the overall trend in the incidence and YLDs of PF in China (15). This study also forecasts the incidence and YLDs of PF over the next 24 years to provide valuable information for the future formulation of better prevention, treatment, and rehabilitation policies for PF in China.

## Materials and methods

### Data sources and case definition

The 2021 Global Burden of Disease (GBD) database covers 371 diseases and injuries from different regions and countries, indicating that the incidence of PF in regions with high sociodemographic index (SDI) is higher than in those with a low SDI. The incidence and YLDs of PF from 1992 to 2021 were obtained from the GBD database using its query exchange tool.^1^ A Bayesian regression algorithm called DisMod-MR was used to assess overall incidence, and YLDs in the 2021 GBD database (16). The data retrieval strategy for this study included the region in China, with categories for male, female, and both sexes. Metrics selected were numbers and rates, with age categories ranging from <5 years up to >95 years. The analysis focused on the incidence and YLDs. Data were compiled and processed using Excel 2021, with a double-data entry and verification approach for accuracy. PF were defined using the International Classification of Diseases Ninth (ICD-9) and Tenth (ICD-10) revision codes.

### Descriptive analysis

In this study, we utilized the number, all ages, and age-standardized incidence rate (ASIR) and age-standardized YLDs rate (ASYR) to evaluate the trends of PF in China from 1992 to 2021. The GBD 2021 age-standardized population model specific to China was matched with these metrics. By introducing this method, allowing for an accurate comparison of incidence and YLDs trends and burden over time.

### Joinpoint regression model

In this study, the JPR model was introduced to analyze the temporal characteristics of disease or injury distribution in terms of incidence and YLDs (17). The model assessed the annual percent change (APC) and average annual percent change (AAPC) for the incidence and YLDs of PF, where APC evaluates changes during a set period, and AAPC represents a comprehensive measure of overall changes over time. An APC > 0 indicates a year-on-year increase, whereas a decrease is indicated otherwise; APC = AAPC suggests the absence of significant turning points in trend.

### Age-period-cohort analysis

The APC model often involves a multiclass model dealing with the effects of age groups, period groups, and birth cohorts. Age effects account for factors, including population aging, on incidence and YLDs. Period effects reflect changes in disease and injury risks owing to objective factors. Cohort effects address how the exposure levels of different birth cohorts to risk factors influence incidence and YLDs. Traditional statistical methods, which cannot eliminate the collinearity between these factors, are bypassed by the intrinsic estimator (IE) method, allowing the APC model to assess the effects of age, period, and cohort separately (18, 19) The APC model is a linear model, represented as follows: In (Refg) = α + Ae + Pf + Cg, where Refg denotes the incidence or mortality rate of PF in g birth cohorts, e refers to age groups, and f represents periods, where Ae, Pf, and Cg represent the effects of age, period, and cohort, respectively.

### Predicting incidence and YLDs

The Nordpred prediction model, based on the APC model, effectively forecasts future trends in the incidence, prevalence, and YLDs of a disease or injury. It considers the relationship between time series and demographic characteristics, including changes in the population structure, disease trends, and generational effects (20). To predict trends in pelvic fracture burden, the Nordpred APC model was used to forecast the incidence and YLDs from 2022 to 2046. The Nordpred software package in R language (version 4.3.1) incorporates dynamic changes in incidence and population structure (21).

### Statistical analysis

Statistical analyses were conducted using R software (4.3.1). The JPR software (4.9.1.0) was used to construct the JPR model, calculating APC, AAPC, and 95% confidence intervals (CI), and to analyze trend changes. The APC model was built using Stata 16.0, and age-specific ratios for each period and cohort through relative risk (RR) were assessed. Predictions were made using the “Nordpred” package in R software (22), and the significance of parameters was estimated using the Wald chi-square test. A *p*-value of <0.05 was considered statistically significant.

## Results

### Description analysis of pelvic fracture prevalence in China

**Figure 1:**
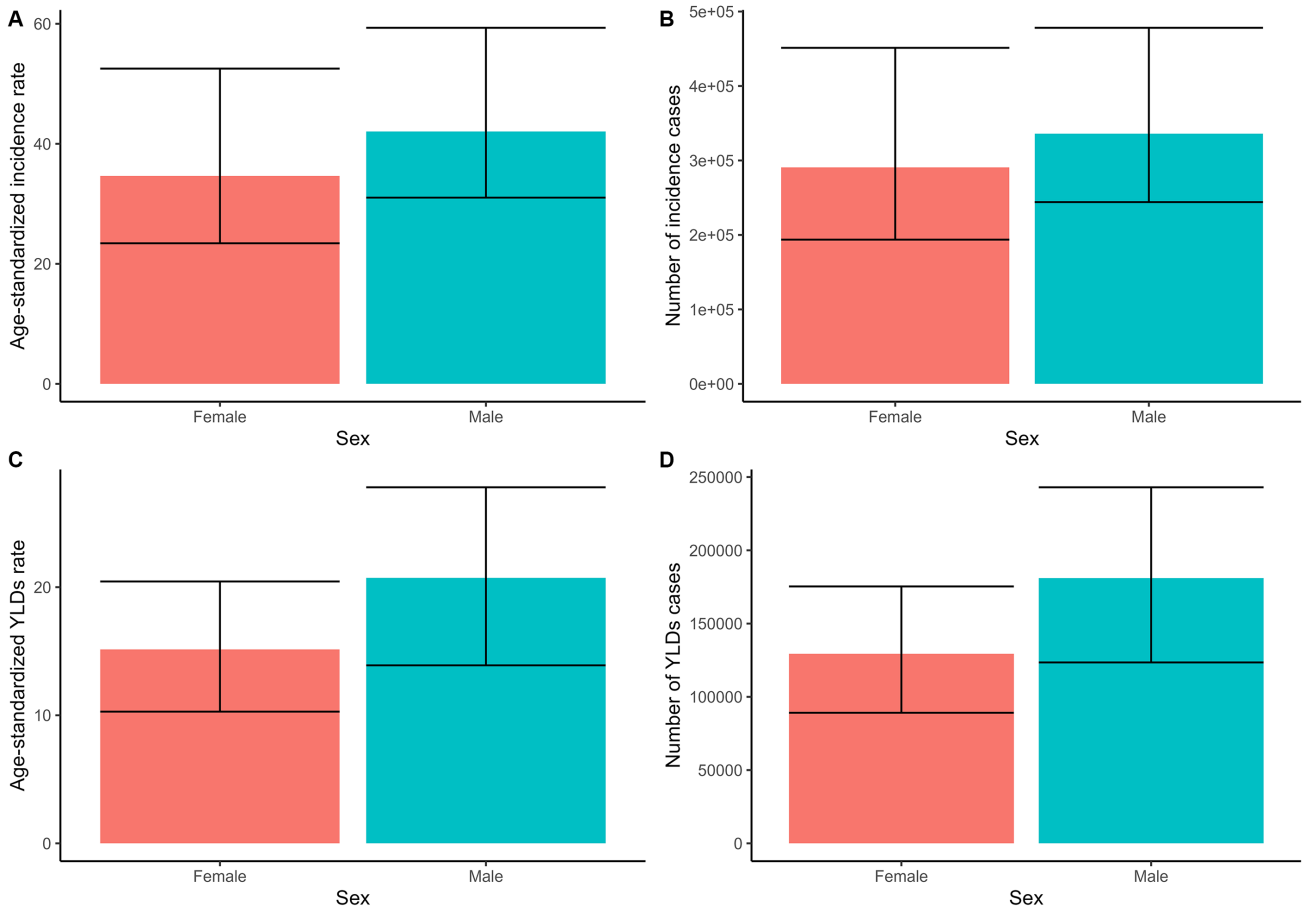
The number and ASR of incidence and YLDs of PF in 2021 by sex. **(A)** ASIR. **(B)** Number of incidence cases. **(C)** ASYR. **(D)** Number of YLDs cases.

**Figure 2:**
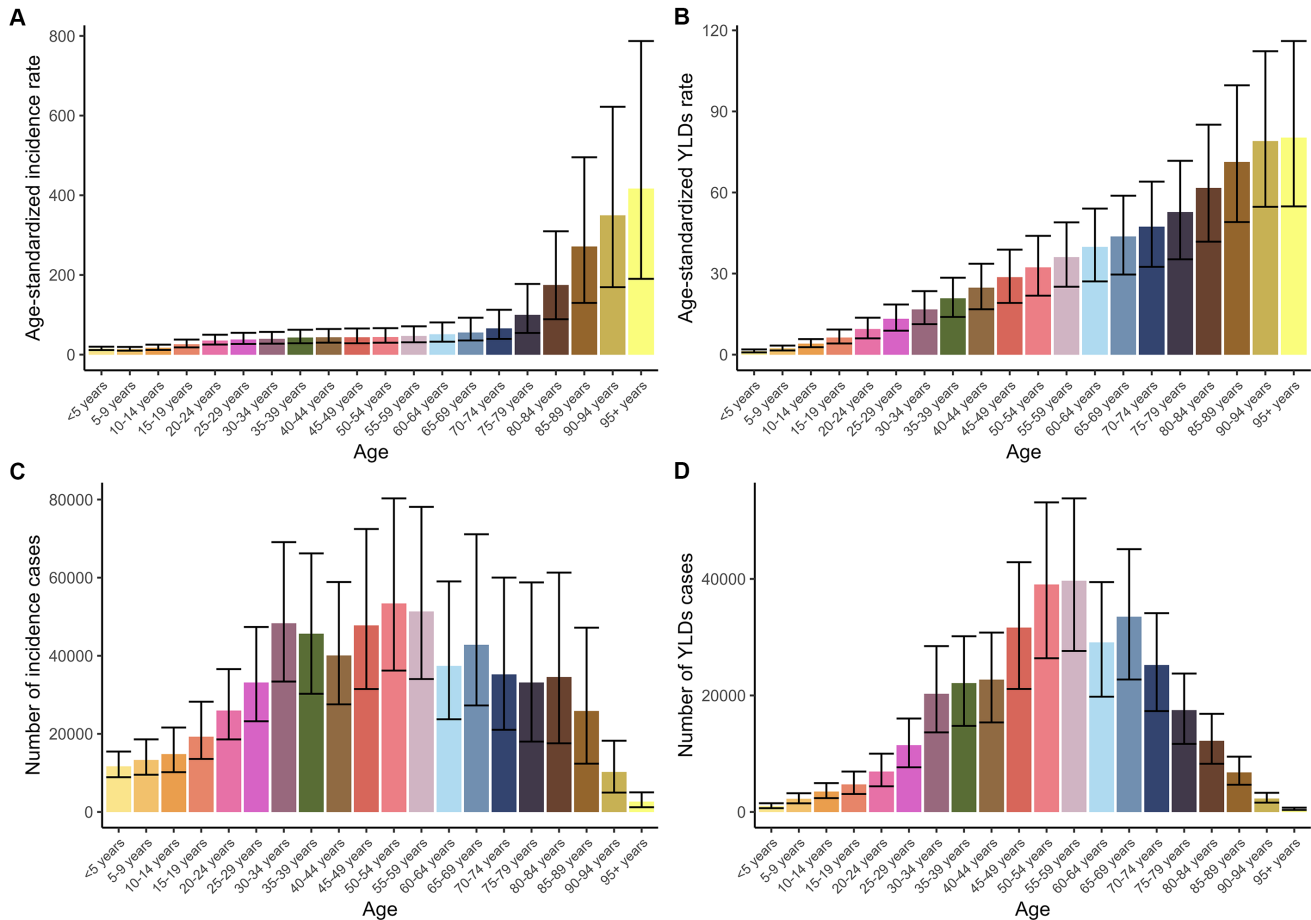
The number and ASR of incidence and YLDs of PF in 2021 by age. **(A)** ASIR. **(B)** ASYR. **(C)** Number of incidence cases. **(D)** Number of YLDs cases.

In 2021, an estimated 0.62 million cases of PF were recorded in China, with approximately 0.33 million cases in men (95% UI: 0.24 to 0.48 million) and 0.29 million cases in women (95% UI: 0.19 to 0.45 million). The total number of YLDs due to PF was about 0.33 million cases, with men accounting for 0.19 million cases (95% UI: 0.13 to 0.26 million) and women for 0.14 million cases (95% UI: 0.1 to 0.19 million). The ASIR of PF was 38.82 per 100,000 people, showing higher rates in men at 42.04 (95% UI: 31.03 to 59.33) compared to women at 34.64 (95% UI: 23.43 to 52.53). The ASYR was 17.97 per 100,000 people, again higher in men at 20.72 (95% UI: 13.9 to 27.8) than in women at 15.15 (95% UI: 10.29 to 20.45; Figure 1; Supplementary Table 1). The peak incidence occurred in the 50–54 age group with 53,397 cases (95% UI: 36,209 to 80,300) and YLDs occurred in 55–59 age group with 39,687 cases YLDs (95% UI: 27,632 to 53,824), respectively (Figure 2; Supplementary Table 1).

**Figure 3:**
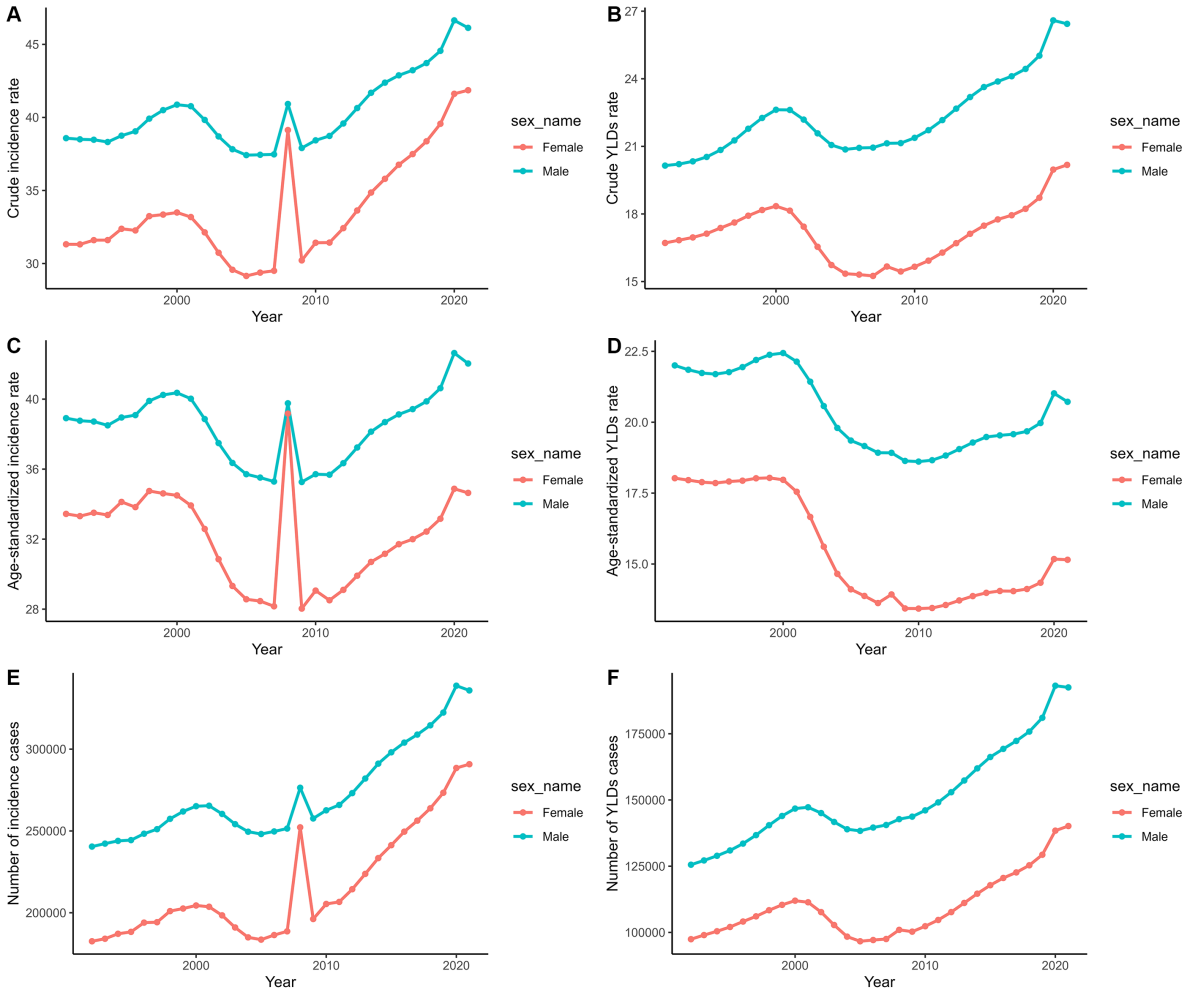
Temporal trends of the number and rate of incidence and YLDs of PF from 1992 to 2021. **(A)** Crude rate of incidence. **(B)** Crude rate of YLDs. **(C)** ASIR. **(D)** ASYR. **(E)** Number of incidence cases. **(F)** Number of YLDs cases.

Specifically, the crude incidence rate (CIR) for men rose from 38.58‰ in 1992 to 46.13‰ in 2021, while for women, it increased from 31.32‰ to 41.86‰. Although similar trends were observed in the ASIR by gender, these rates experienced mild fluctuations. Over the same period, the crude YLDs (CYR) for PF in men increased from 20.15‰ to 26.44‰, and in women from 16.71‰ to 20.18‰. However, the increases in the ASYR were less pronounced (Figure 3; Supplementary Table 2).

### Trends in the incidence and YLDs of PF in China from 1992 to 2021

**Figure 4:**
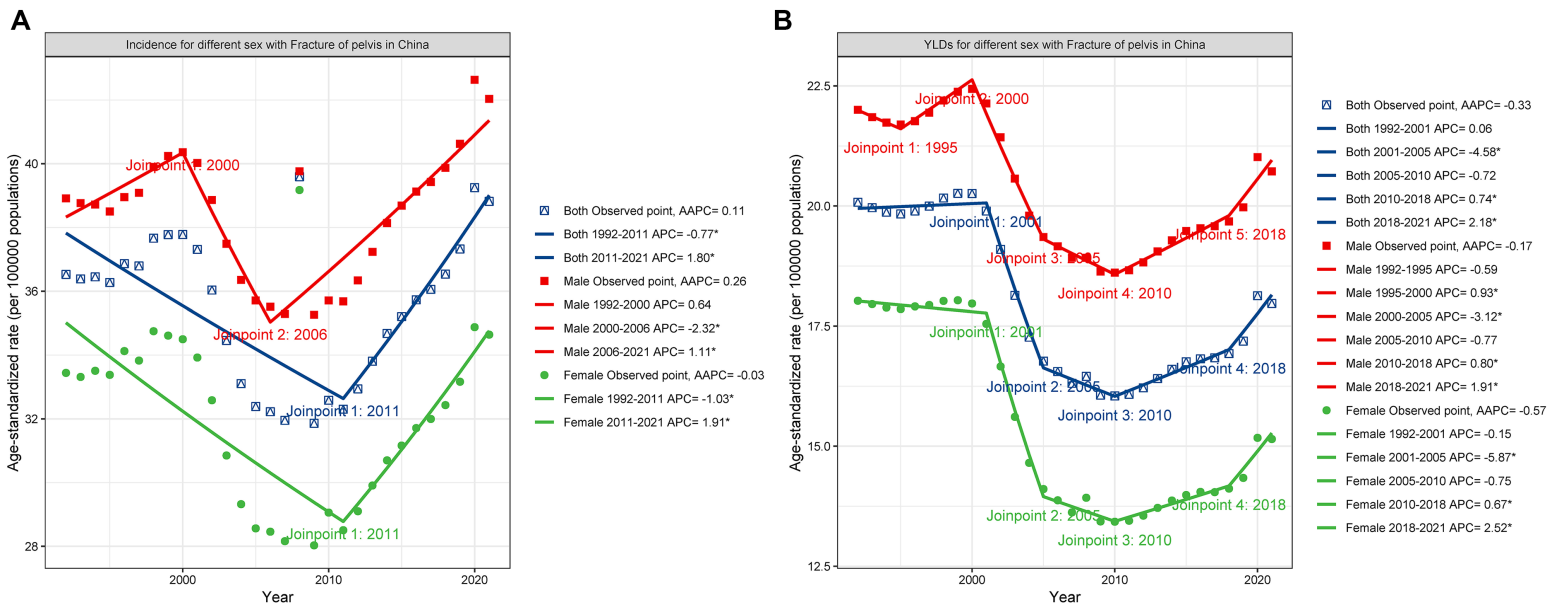
Joinpoint regression analyze the trends ASR for both sexes of PF from 1992 to 2021 in China. **(A)** APCs of ASIR for overall, males, and females. **(B)** APCs of ASYR for overall, males, and females.

Joinpoint regression analysis revealed a significant decline in the overall incidence of PF in China from 1992 to 2011. This declining trend was also observed among females. Among males, a slight increase in incidence was noted from 1992 to 2000, followed by a significant decline from 2000 to 2006, and then a significant upward trend from 2006 to 2021 (Figure 4A). Joinpoint regression analysis indicated a relatively stable trend in the overall YLDs of PF in China from 1992 to 2001, followed by a significant decline from 2001 to 2005, and an upward trend from 2010 to 2018 and 2018 to 2021. This pattern was also observed among females. For males, the trend was more complex, with a slight upward trend from 1995 to 2000, a significant decline from 2000 to 2005, and significant upward trends from 2010 to 2018 and 2018 to 2021 (Figure 4B). From 1992 to 2021, the AAPC values for incidence and YLDs were 0.11 and 0.33%, respectively, with YLDs changes being statistically significant (*p* = 0.019; Table 1). The AAPC values for incidence and YLDs in males were 0.26% and −0.17%, respectively, both the incidence and YLDs change were not statistically significant. For females, the AAPC values for incidence and YLDs were −0.03% and −0.57%, respectively, with YLDs changes being statistically significant (*p* < 0.001; Table 1).

**Table 1:** Joinpoint regression analysis: the APC and AAPC in the incidence and YLDs of PF in China from 1992 to 2021.

Segments	Incidence							YLDs						
	Year	APC(%)	95%CI	*p*-value	AAPC	95%CI	*p*-value	Year	APC(%)	95%CI	*p*-value	AAPC	95%CI	*p*-value
Both														
	1992 ~ 2011	−0.77	−1.17 to −0.38	0.001	0.11	−0.37 to 0.58	0.661	1992 ~ 2001	0.06	−0.18 to 0.3	0.582	−0.33	−0.6 to −0.05	0.019
	2011 ~ 2021	1.8	0.55 to 3.06	0.006				2001 ~ 2005	−4.58	−5.83 to −3.32	0.001			
	/	/	/	/				2005 ~ 2010	−0.72	−1.55 to 0.11	0.084			
	/	/	/	/				2010 ~ 2018	0.74	0.39 to 1.09	0.001			
	/	/	/	/				2018 ~ 2021	2.18	0.85 to 3.53	0.003			
Male														
	1992 ~ 2000	0.64	−0.29 to 1.59	0.169	0.26	−0.21 to 0.74	0.276	1992 ~ 1995	−0.59	−1.81 to 0.65	0.32	−0.17	−0.45 to 0.12	0.25
	2000 ~ 2006	−2.32	−4.11 to −0.51	0.015				1995 ~ 2000	0.93	0.13 to 1.73	0.026			
	2006 ~ 2021	1.11	0.73 to 1.5	0.001				2000 ~ 2005	−3.12	−3.89 to −2.35	0.001			
	/	/	/	/				2005 ~ 2010	−0.77	−1.56 to 0.02	0.054			
	/	/	/	/				2010 ~ 2018	0.8	0.47 to 1.14	0.001			
	/	/	/	/				2018 ~ 2021	1.91	0.64 to 3.19	0.006			
Female														
	1992 ~ 2011	−1.03	−1.54 to −0.51	0.001	−0.03	−0.63 to 0.59	0.935	1992 ~ 2001	−0.15	−0.4 to 0.09	0.207	−0.57	−0.85 to −0.29	<0.001
	2011 ~ 2021	1.91	0.32 to 3.52	0.02				2001 ~ 2005	−5.87	−7.14 to −4.59	0			
	/	/	/	/				2005 ~ 2010	−0.75	−1.61 to 0.11	0.084			
	/	/	/	/				2010 ~ 2018	0.67	0.31 to 1.04	0.001			
	/	/	/	/				2018 ~ 2021	2.52	1.12 to 3.93	0.001			
														

AAPC, average annual percent change presented for full period; APC, annual percent change; CI, confidence interval.

### APC model analysis of pelvic fracture incidence in china

**Figure 5:**
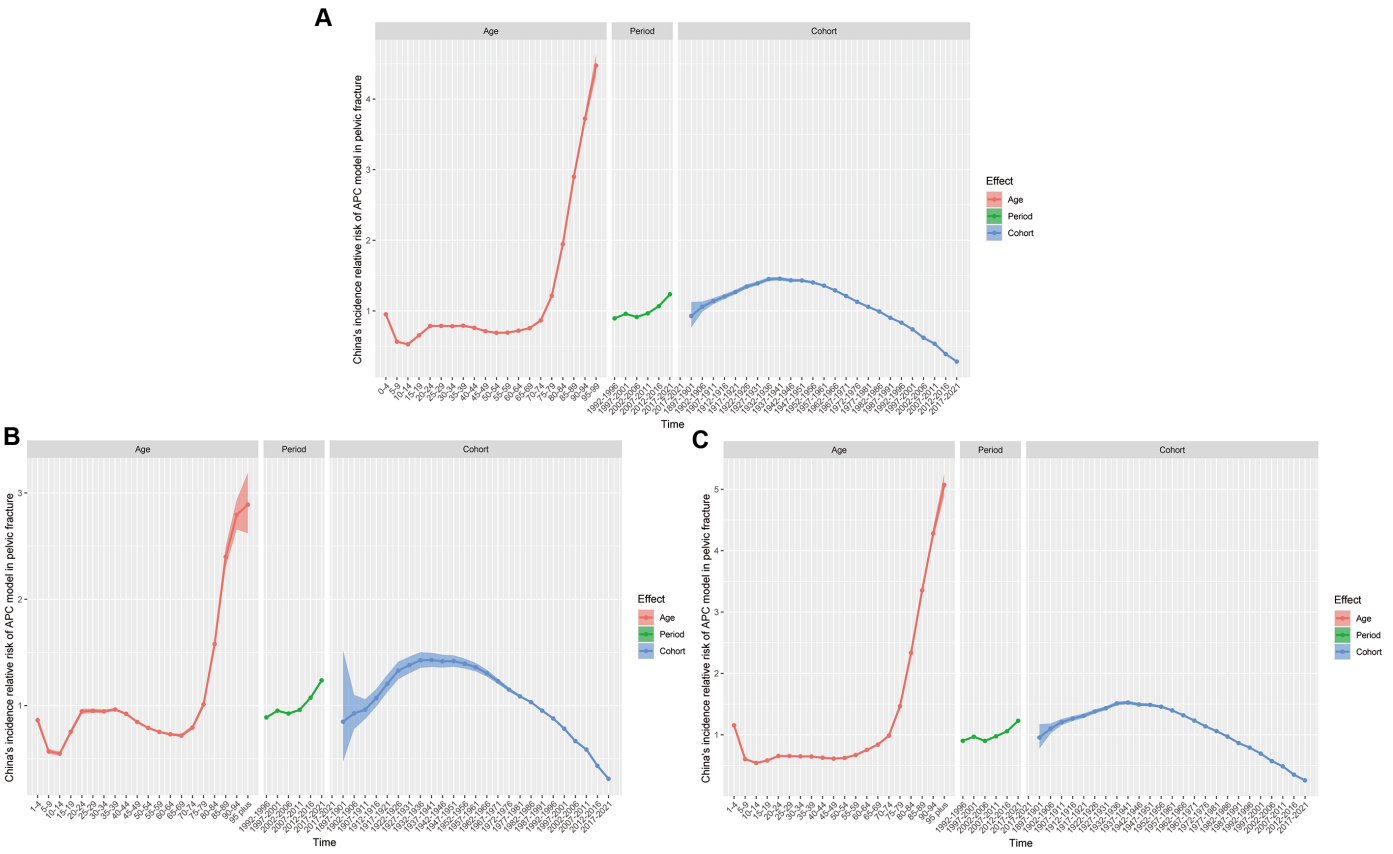
Estimated relative risks of age, period, and cohort effects on the incidence of PF. **(A)** RR of both sexes. **(B)** RR of male. **(C)** RR of female.

The results on the age effect analysis showed that the risk of pelvic fracture in the overall population increases with age, with the lowest risk in the group aged 10–14 years (RR = 0.53, 95% CI, 0.52 to 0.54) and the highest risk in those aged >95 years (RR = 4.47, 95% CI, 4.33 to 4.62). The incidence in males first decrease, and then totally increases again, with the lowest risk in the group aged 10–14 years (RR = 0.55, 95% CI, 0.53 to 0.57) and the highest risk in the group aged >95 years (RR = 2.89, 95% CI, 2.62 to 3.19). For females, the incidence trend also showed an initial decline, followed by a gradual increase, with the lowest risk in the group aged 10–14 years (RR = 0.54, 95% CI, 0.53 to 0.55) and the highest risk in those aged >95 years (RR = 5.07, 95% CI, 4.9 to 5.25). The results of the period effect analysis indicate that the risk of PF in the overall, male, and female populations showed a trend of initially rising, declining, and then rising again, with the lowest cohort effects in 1992–1996 for the overall and male populations (RR = 0.9, 95% CI, 0.89 to 0.9; RR = 0.9, 95% CI, 0.88 to 0.9) and in 2002–2006 for female populations (RR = 0.9, 95% CI, 0.9 to 0.91). The highest risk occurs in 2015–2019 for the overall, male, and female populations (RR = 1.24, 95% CI, 1.23 to 1.24; RR = 1.24, 95% CI, 1.22 to 1.25; and RR = 1.23, 95% CI, 1.22 to 1.24, respectively). The results of the cohort analysis show a upward trend firstly, followed by a significant declining trend in the risk of PF over time in the overall, male, and female populations, with the highest risk in the 1937–1941 cohort for the overall, male, and female populations (RR = 1.46, 95% CI, 1.43 to 1.48; RR = 1.43, 95% CI, 1.36 to 1.5; and RR = 1.53, 95% CI, 1.5 to 1.56, respectively) and the lowest risk in the 2017–2021 cohort for the overall, male, and female populations (RR = 0.28, 95% CI, 0.28 to 0.29; RR = 0.31, 95% CI, 0.3 to 0.32; and RR = 0.26, 95% CI, 0.25 to 0.27), respectively (Figure 5; Supplementary Table 3).

### APC model analysis of YLDs of PF in China

**Figure 6:**
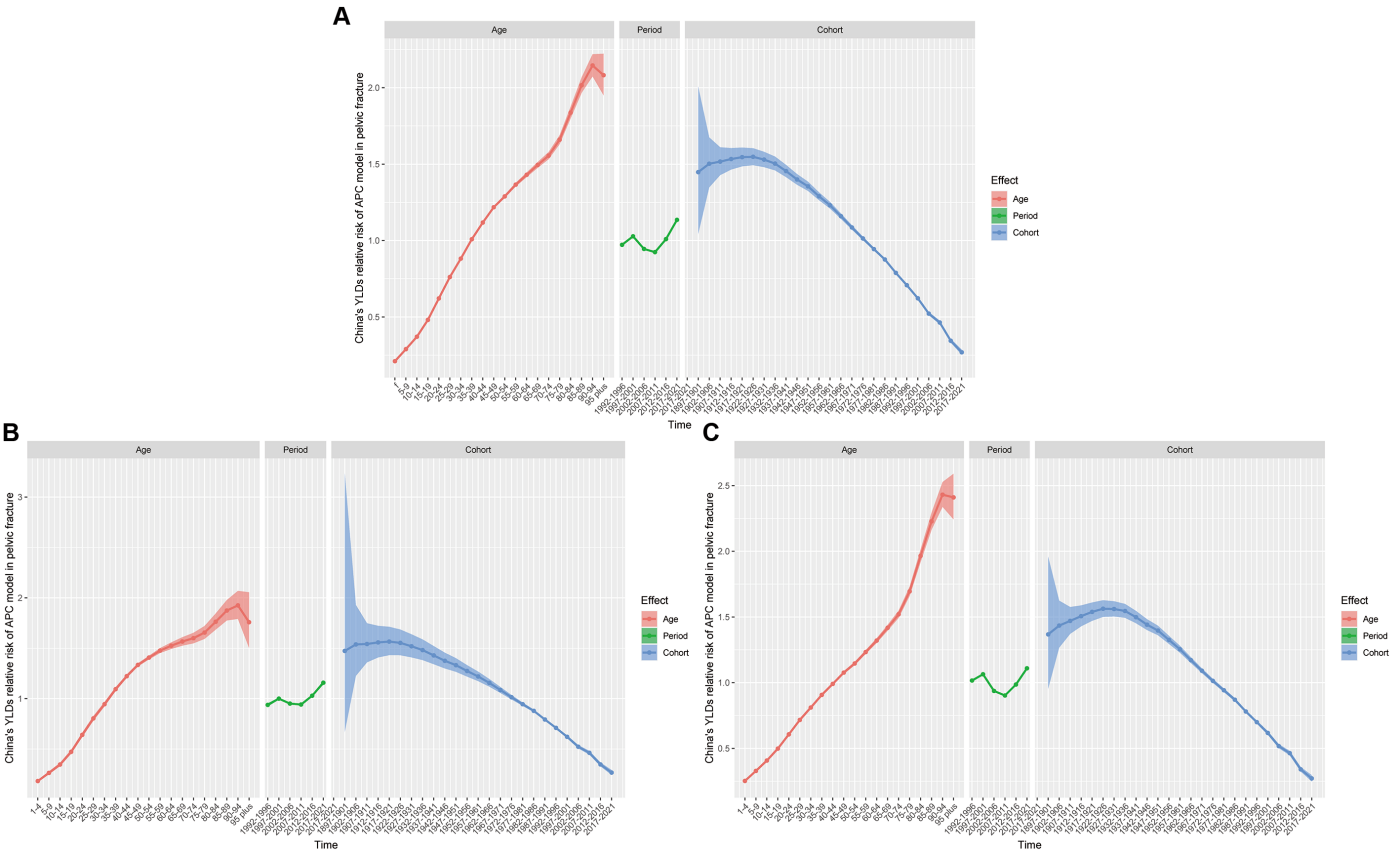
Estimated relative risks of age, period, and cohort effects on the YLDs of PF. **(A)** RR of both sexes. **(B)** RR of male. **(C)** RR of female.

The results of the age effect analysis for YLDs of PF show a trend of increasing risk with age, with the lowest risk in the group aged 1–4 years (RR = 0.21, 95% CI, 0.2 to 0.22) and the highest risk in those aged 90–94 years (RR = 2.15, 95% CI, 2.07 to 2.22). The lowest risk for males was noted in the group aged 1–4 years (RR = 0.18, 95% CI, 0.17 to 0.2) and the highest risk in the group aged 90–94 years (RR = 1.93, 95% CI, 1.79 to 2.07). For females, the lowest risk was noted in the group aged 1–4 years (RR = 0.25, 95% CI, 0.24 to 0.26), and the highest risk was observed in those aged 90–94 years (RR = 2.43, 95% CI, 2.34 to 2.53). The results of the period effect analysis for YLDs show a trend of initially rising, followed by a decline trend, and then rising again for the overall, male, and female populations, with the lowest risk in 2007–2011 (RR = 0.92, 95% CI, 0.92 to 0.93; RR = 0.94, 95% CI, 0.94 to 0.95; and RR = 0.9, 95% CI, 0.9 to 0.91, respectively). The results of the cohort analysis for YLDs showed an initial rise followed by a gradual decline for the overall, male and female populations. The highest risk was noted in the 1922–1926 cohort for the overall, and female populations (RR = 1.55, 95% CI, 1.49 to 1.6; and RR = 1.56, 95% CI, 1.5 to 1.63, respectively), and in the 1917–1921 cohort for male (RR = 1.57, 95% CI, 1.43 to 1.71). The lowest risk in the 2017–2021 cohort for the overall, and female populations (RR = 0.27, 95% CI, 0.25 to 0.29; RR = 0.27, 95% CI, 0.24 to 0.29; and RR = 0.27, 95% CI, 0.25 to 0.3), respectively (Figure 6; Supplementary Table 4).

### Forecasting the incidence and YLDs of PF in China from 2022 to 2046

The ASIR and ASYR of PF in China from 2022 to 2046 are projected to show a trend of increase followed by a gradual decline, with a turning point around 2029. From 2022 to 2029, the ASIRs for the overall, male, and female populations are expected to increase from 38.8 per 100,000, 42.39 per 100,000, and 34.54 per 100,000 in 2022 to 40.88 per 100,000, 44.64 per 100,000, and 36.27 per 100,000 in 2029, representing increases of approximately 5.36, 5.31, and 5.01%, respectively. The ASYRs’ turning point is around 2032, from 17.85 per 100,000, 20.74 per 100,000, and 14.94 per 100,000 in 2022 to 18.64 per 100,000, 21.66 per 100,000, and 15.59 per 100,000 in 2034 represent increases of approximately 4.43, 4.44, and 4.35%, respectively.

**Figure 7:**
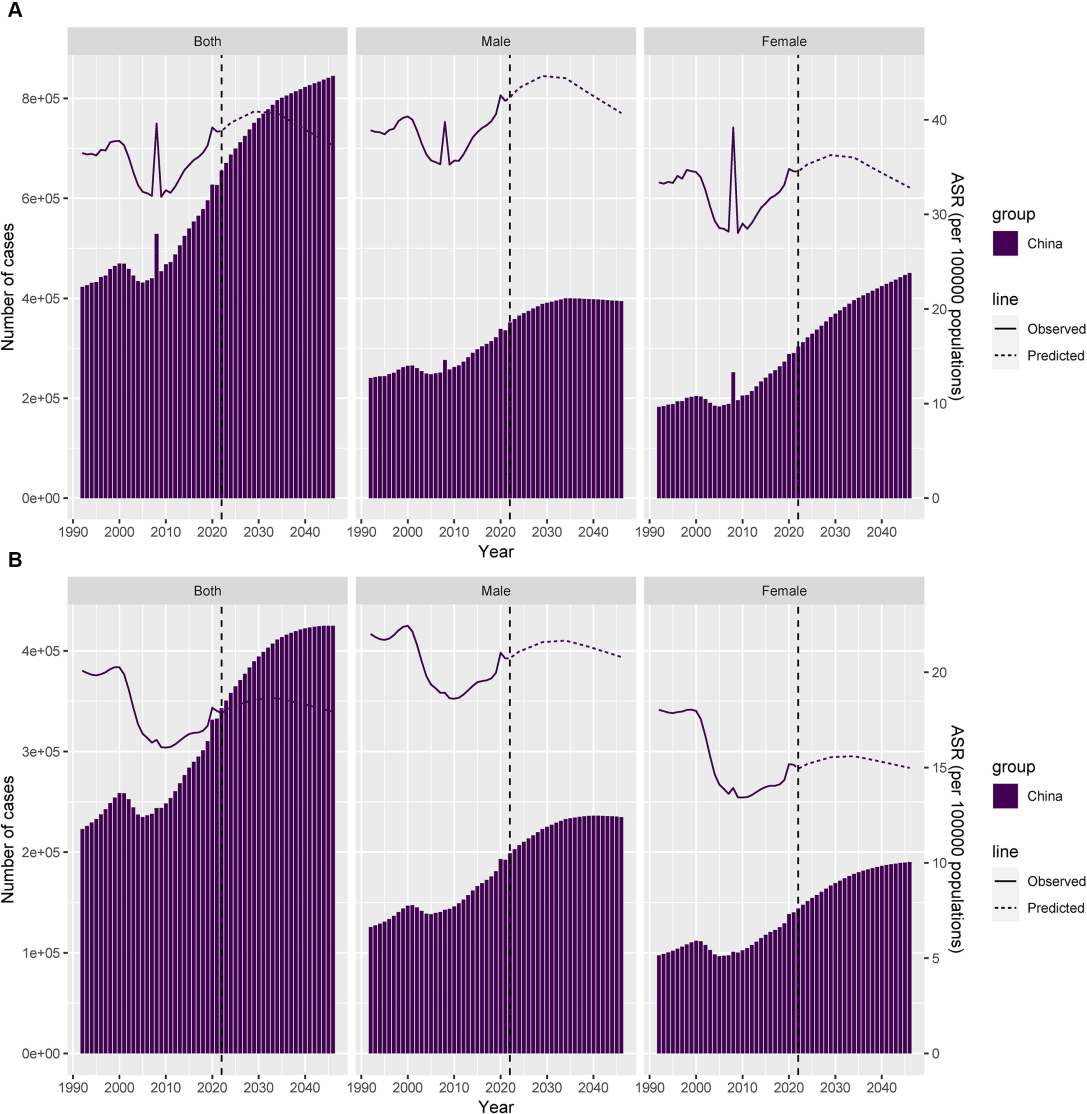
Predicted trends of PF incidence and YLDS in China over 24 years (2022–2046). **(A)** Number and ASR of incidence trends. **(B)** Number and ASR of YLDs trends.

After approximately 2029, the incidence of PF in China is expected to gradually decline, with the ASIRs for the overall, male, and female populations decreasing from 40.88 per 100,000, 44.64 per 100,000, and 36.27 per 100,000 in 2029 to 37.3 per 100,000, 40.72 per 100,000, and 32.83 per 100,000 in 2046, representing declines of approximately 8.76, 8.78, and 9.48%, respectively. The ASYRs from 18.64 per 100,000, 21.66 per 100,000, and 15.59 per 100,000 in 2034 to 17.93 per 100,000, 20.8 per 100,000, and 14.98 per 100,000 in 2046 represent declines of approximately 3.81, 3.97, and 3.91%, respectively. Both the incidence and YLDs number of cases were projected to increase in the following 24 years. In 2008, a minor peak was observed in both incidence and YLDs, which was associated with a catastrophe earthquake in Wenchuan County, Sichuan Province, China (Figure 7; Supplementary Table 5).

## Discussion

Our study found that in 2021, there were 0.63 million new cases of PF in China, along with 0.33 million cases of YLDs. The annual increase rates of incidence and YLDs for both men and women were statistically significant. The RR of incidence and YLDs increased with age. Period effects showed an initial rise followed by a decline in risk. Cohort effects revealed a slight upward trend followed by a significant reduction in risk for birth cohorts in both incidence and YLDs. It is projected that starting from 2029 and 2034, the incidence and YLDs of pelvic fractures in China will gradually decrease, respectively. PF are often seen in patients with multiple injuries caused by high-velocity impacts, such as traffic accidents and falls from a height. Data show that PF occur in 20–37 per 100,000 populations (23). In China, the number of severe multiple injuries has been trending downward, which may due to increased awareness of traffic safety and improved population education. However, multiple injuries still pose a serious threat to public health and safety, particularly with PF. Thus, a deeper investigation into the trends in the incidence of PF and YLDs will aid in refining diagnostic and treatment strategies and assist policymakers in making effective decisions.

This study found that about in 2010 as the turning point, the incidence and YLDs of PF in China significantly declined, likely closely related to public health interventions and enhanced traffic safety awareness. Improvements in medical service quality and standardized treatment for PF have enriched treatment concepts, improved treatment efficiency, and reduced disability and mortality rates (24). However, since 2010 the incidence and YLDs of PF has been increasing annually, which was possibly related to the pace of modernization and the accelerating trend of population aging in China. Older people are more susceptible to fractures caused by osteoporosis, especially in females, possibly related to the higher incidence of osteoporosis in older women (25).

The APC model study identified APC effects for PF in China. The age effect showed that children have a lower risk of PF, mainly because they are less exposed to high-risk factors. The incidence of PF is higher among older adults, which is related to osteoporosis where even low-energy impacts can cause fractures (26, 27). The period effect indicated a decrease in the risk of PF during the 1990s; however, a gradual increase was noted since the beginning of the 21st century, which may be related to population aging and improved diagnostic levels (28, 29). Notably, from the 1997–2001 to 2007–2011 periods combined showed a significant decrease in risk levels, which may be closely related to increased public health attention and the implementation of improved policies. The cohort effect totally showed a general declining trend in the risk of PF with each birth cohort, which is likely linked to improvements in medical standards and the vigorous development of prevention and treatment measures. Notably, the incidence of pelvic fractures is highest among cohorts born around 1932–1936 and 1937–1941. Given their current age, this aligns with the trend of population aging, indicating that this group is particularly susceptible to fragility-related pelvic fractures. However, these results are merely based on data organized by the GBD database, and the actual situation may differ, necessitating targeted data collection and analysis in the future.

Trend predictions indicate that the ASIRs and ASYRs show a trend of initially rising significantly and then gradually declining, with a turning point around 2029 and 2034, respectively. From 2022 to 2029 and 2022 to 2034, the ASIR and ASYRs for the overall, male, and female populations increased, respectively. This trend could be related to various factors. Data show that in 2010s, Chinese population aged >60 years was approximately 254 million; however, this number is expected to increase to 402 million by 2040, accounting for approximately 28% of the total population (30, 31), significantly increasing the overall risk of PF. After 2029 and 2034 for ASIR and ASYR, the gradual decline of PF may be related to improvements in medical conditions and preventive measures. As the population ages further, more strategies for preventing osteoporosis are likely to be disseminated, effectively reducing the incidence of PF in older people. Notably, this study is based on historical data and current trends and does not fully consider potential changes in new medical technologies and policy directions that might affect future trends. Therefore, future research must consider these potential trend-affecting factors comprehensively.

In conclusion, the changes in the incidence and YLDs of PF in China exhibit complex and variable characteristics, with certain correlations between the overall population and sex differences, yet each displaying distinct traits. The occurrence of PF among young and older populations both show relative peaks; the former was likely closely associated with factors such as traffic accidents and falls at construction sites, and the latter possibly related to population aging and osteoporosis-related fragility fractures. This study also found that the outcomes of pelvic fractures exhibit a certain lag in YLDs compared to incidence rates, beyond the inherent epidemiological statistical characteristics. Incidence rates are more immediate indicators, reflecting the incidence and spread of diseases. In contrast, YLDs capture the long-term impact of diseases and thus generally exhibit a lag relative to incidence rates. Based on these results, the development of primary prevention efforts, enhanced production safety publicity and supervision, improved traffic safety awareness, and enhanced traffic safety regulations are recommended. Raising awareness of osteoporosis prevention among older adults, increasing public health education, and promoting good dietary and hygiene habits are essential to comprehensively reduce the incidence of PF in China.

## Data availability statement

The original contributions presented in the study are included in the article/Supplementary material, further inquiries can be directed to the corresponding author.

## Author contributions

QC: Conceptualization, Data curation, Formal analysis, Investigation, Project administration, Software, Supervision, Writing – original draft, Writing – review & editing. TL: Funding acquisition, Investigation, Software, Writing – original draft, Writing – review & editing. HD: Formal analysis, Methodology, Writing – original draft. GH: Formal analysis, Methodology, Writing – original draft. DD: Conceptualization, Project administration, Supervision, Writing – review & editing. JY: Conceptualization, Project administration, Supervision, Writing – review & editing.

## Footnotes

^1^ https://vizhub.healthdata.org/gbd-results/
