# Supplementary material for: Age–period–cohort analysis of epidemiological trends in pelvic fracture in China from 1992 to 2021 and forecasts for 2046
Source: Front Public Health. 2024 Jul 8;12:1428068. doi: 10.3389/fpubh.2024.1428068 (PMC11260792; doi:10.3389/fpubh.2024.1428068)
Supplement: Supplementary file 1 [file Data_Sheet_1.docx]

Supplementary Material

**Age–period–cohort analysis of epidemiological trends in pelvic fracture in China from 1992 to 2021 and forecasts for 2046**

**Qingsong Chen1,2#, Tao Li1#, Hong Ding3, Guangbin Huang1, Dingyuan Du1, Jun Yang1***

*** Correspondence:** Jun Yang, E-mail address: Yangjun1970399@sina.com

# Supplementary Figures and Tables

For more information on Supplementary Material and for details on the different file types accepted, please see [here](https://www.frontiersin.org/guidelines/author-guidelines#supplementary-material).

## Supplementary Tables

**Supplementary table 1** Temporal trends of incidence and YLDs from 1992 to 2021 by age and sex.

**Supplementary table 2** PF crude and age-standardized rate of incidence and YLDs trends in China from 1992 to 2021.

**Supplementary table 3** Age–period–cohort effects of PF incidence in China.

**Supplementary table 4** Age–period–cohort effects of PF YLDs in China.

**Supplementary table 5** Forecasted counts and rates of PF in China from 2022 to 2046.
